# Contribution of the ankle-brachial index to improve the prediction of coronary risk: The ARTPER cohort

**DOI:** 10.1371/journal.pone.0191283

**Published:** 2018-01-16

**Authors:** Rosa Forés, Maria Teresa Alzamora, Guillem Pera, José Miguel Baena-Díez, Xavier Mundet-Tuduri, Pere Torán

**Affiliations:** 1 Centre d’Atenció Primària Riu Nord- Riu Sud Santa Coloma de Gramenet, Direcció d’Atenció Primària Barcelonés Nord i Maresme, Institut Català de la Salut, Barcelona, Spain; 2 Unitat de Suport a la Recerca Metropolitana Nord, Institut Universitari d’Investigació en Atenció Primària Jordi Gol (IDIAP Jordi Gol), Mataró, Spain; 3 Universitat Autònoma de Barcelona, Bellaterra (Cerdanyola del Vallès), Spain; 4 Centre d’Atenció Primària La Marina, Direcció d’Atenció Primària Barcelona Ciutat, Institut Català de la Salut, Barcelona, Spain; 5 Institut Universitari d’Investigació en Atenció Primària Jordi Gol (IDIAP Jordi Gol), Barcelona, Spain; 6 Unitat de Suport a la Recerca Barcelona, Institut Universitari d’Investigació en Atenció Primària Jordi Gol (IDIAP Jordi Gol), Barcelona, Spain; Spectrum Health, UNITED STATES

## Abstract

**Background:**

The different cardiovascular risk prediction scales currently available are not sufficiently sensitive.

**Aim:**

The aim of the present study was to analyze the contribution of the ankle-brachial index (ABI) added to the Framingham and REGICOR risk scales for the reclassification of cardiovascular risk after a 9-year follow up of a Mediterranean population with low cardiovascular risk.

**Design and setting:**

A population-based prospective cohort study was performed in the province of Barcelona, Spain.

**Method:**

A total of 3,786 subjects >49 years were recruited from 2006–2008. Baseline ABI was performed and cardiovascular risk was calculated with the Framingham and REGICOR scales. The participants were followed until November 2016 by telephone and review of the clinical history every 6 months to confirm the possible appearance of cardiovascular events.

**Results:**

2,716 individuals participated in the study. There were 126 incidental cases of first coronary events (5%) during follow up. The incidence of coronary events in patients with ABI <0.9 was 4-fold greater than that of subjects with a normal ABI (17.2/1,000 persons-year versus 4.8/1,000 persons-year). Improvement in the predictive capacity of REGICOR scale was observed on including ABI in the model, obtaining a net reclassification improvement of 7% (95% confidence interval 0%-13%) for REGICOR+ ABI. Framingham + ABI obtained a NRI of 4% (-2%-11%).

**Conclusion:**

The results of the present study support the addition of the ABI as a tool to help in the reclassification of cardiovascular risk and to confirm the greater incidence of coronary events in patients with ABI < 0.9.

## Introduction

In the last decades death by ischaemic heart disease standardized by age has decreased in most regions of the world, particularly in the most developed countries [1]. Nonetheless, cardiovascular disease continues to be the main cause of death in these countries.

One of the challenges of public healthcare involves reducing the incidence of this disease. Over the years this has led to the development of different scales of cardiovascular risk [2,3] adapted to the different populations studied to identify the individuals most susceptible to presenting cardiovascular events. These cardiovascular risk scales have an acceptable predictive capacity in subjects classified as having high risk. The problem is with individuals classified with low and intermediate risk which involves most of the population. Indeed, the largest proportion of cardiovascular events are produced in these subjects making it a priority to correctly classify the individuals who would most benefit from intensive treatment of the risk factors implicated in its appearance. On the other hand, new markers allowing better discrimination of the grade of cardiovascular risk have been studied [4,5], while other studies have been aimed at the identification of biomarkers [6] and the detection of subclinical arteriosclerosis by the measurement of carotid intima-media thickness or the determination of intracoronary calcium [4,7,8]. However, these tests cannot be performed in primary care centers.

Determination of the ankle-brachial index (ABI) is a simple valid diagnostic test to detect peripheral artery disease (PAD) with stenosis greater than 50% in the arteries of the lower extremities [9]. It is well accepted by patients and allows non-invasive identification of subclinical arteriosclerosis. Numerous studies have shown an increase in the risk of cardiovascular morbimortality in patients with an ABI < 0.9 [10–14]. Likewise, other studies of cohorts in different populations have evaluated the change in predictive capacity of cardiovascular risk scales with the addition of the ABI in the calculation [15–23], albeit with contradictory results.

In 2011 our group published a cross-sectional study on the reclassification of cardiovascular risk adding low ABI to the risk scales [24].

The aim of the present study was to evaluate the contribution of the addition of ABI values to the Framingham [25] and REGICOR [3] risk scales in the reclassification of cardiovascular risk after 9 years of follow up of a Mediterranean population with low cardiovascular risk.

## Material and methods

This study was approved by the local Ethics Committee (IDIAP Jordi Gol Foundation of Investigation in Primary Care and Instituto de Salud Carlos III). Informed written consent was obtained from all the participants. Likewise, the recommendations of the World Medical Association Declaration of Helsinki were followed.

The methodology of the prospective, population cohort ARTPER study has been described previously [26,27]. The first phase was carried out from September 2006 to June 2008 in order to determine the prevalence of PAD in our setting. During this period a total of 3,786 subjects over 49 years of age ascribed to 28 primary care centers in Barcelona were included. Recruitment was performed by simple randomization using the database of the population ascribed to the primary care centers participating in the study (more in depth and updated data source than the census) [28]. Participation was 63%.

Two previously trained registered nurses performed the ABI in all the participants under standardized conditions using a portable Doppler device (Mini-DopplexD900-Ps, Huntleigh Healthcare, 8 MHz). The ABI of each lower extremity was calculated by dividing the highest value of systolic blood pressure (SBP) of the posterior tibial or dorsalis pedis arteries by the highest SBP measured in both humeral arteries. A patient was considered to have PAD when the ABI was < 0.9 and arterial calcification with an ABI ≥ 1.4.

The demographic variables of age and sex were collected as were data related to smoking, clinical history of arterial hypertension, hypercholesterolemia, diabetes mellitus, acute myocardial infarction, angina, stroke, transient ischaemic attack, blood pressure, total cholesterol and high density lipoprotein and low density lipoprotein values, triglycerides, glucose and glycosylated haemoglobin in diabetic patients, prescription of antihypertensive, hypolipemia or hypoglycaemic treatment and the calculation of cardiovascular risk using the Framingham [25] and REGICOR [3] equations. The latter is an adaptation of the Framingham score which has been calibrated and validated for the Spanish population [3,29,30]. Patients with previous cardiovascular events (acute myocardial infarction, angina, stroke, transient ischaemic attack, symptomatic abdominal aorta aneurysm, and vascular surgery (coronary, intracranial and extracranial) were excluded from the study. The participants were classified into three categories for each of the risk tables: a) low risk: Framingham < 10%, REGICOR < 5%; b) intermediate risk: Framingham 10–19.9%, REGICOR 5–9.9%; c) high risk: Framingham ≥ 20%, REGICOR ≥10%. The two scales evaluated the appearance of myocardial infarction, angina and coronary revascularization. The patients were followed until November 2016 by telephone call and if a possible cardiovascular event was detected (myocardial infarction, angina, stroke, transient ischaemic attack and vascular coronary surgery) by this method it was confirmed by a group of general practitioners through the review of the electronic medical records, computerized clinical history, personal or telephone interview with the general practitioner in charge of the patient, the emergency departments and emergency paramedical services, and the mortality records. Finally, all the events were checked by a medical committee the members of which carry out routine clinical practice. If no records were found to be able to confirm an event this was not included in the study.

### Statistical analysis

Continuous variables are expressed with mean and standard deviation and categorical variables with frequencies and percentages. Incidence rates are expressed per 1000 person-years and with their 95% confidence intervals. Differences between normal (≥ 0.9) and pathological (< 0.9) ABI subjects were assessed using the t-test for continuous variables, chi-square test for categorical variables and Poisson models for incidence rates. Only the first incident cardiac event was considered. Hazard ratios (HR) of having a cardiac event were computed separately for the Framingham and REGICOR risk tables using Cox models with pathological ABI and the risk table categories as mutually adjusted explanatory variables. Interaction tests between pathological ABI and the risk tables were computed via likelihood ratio tests. The performance of the models using only the Framingham or REGICOR tables with the addition of pathological ABI into the models was compared using the Akaike Index Criteria [31], Harrell’s C [32] and the Net Reclassification Index (NRI) [33]. All tests were bilateral using 0.05 significance. Statistical analysis was performed with Stata v14.

## Results

**Fig 1:**
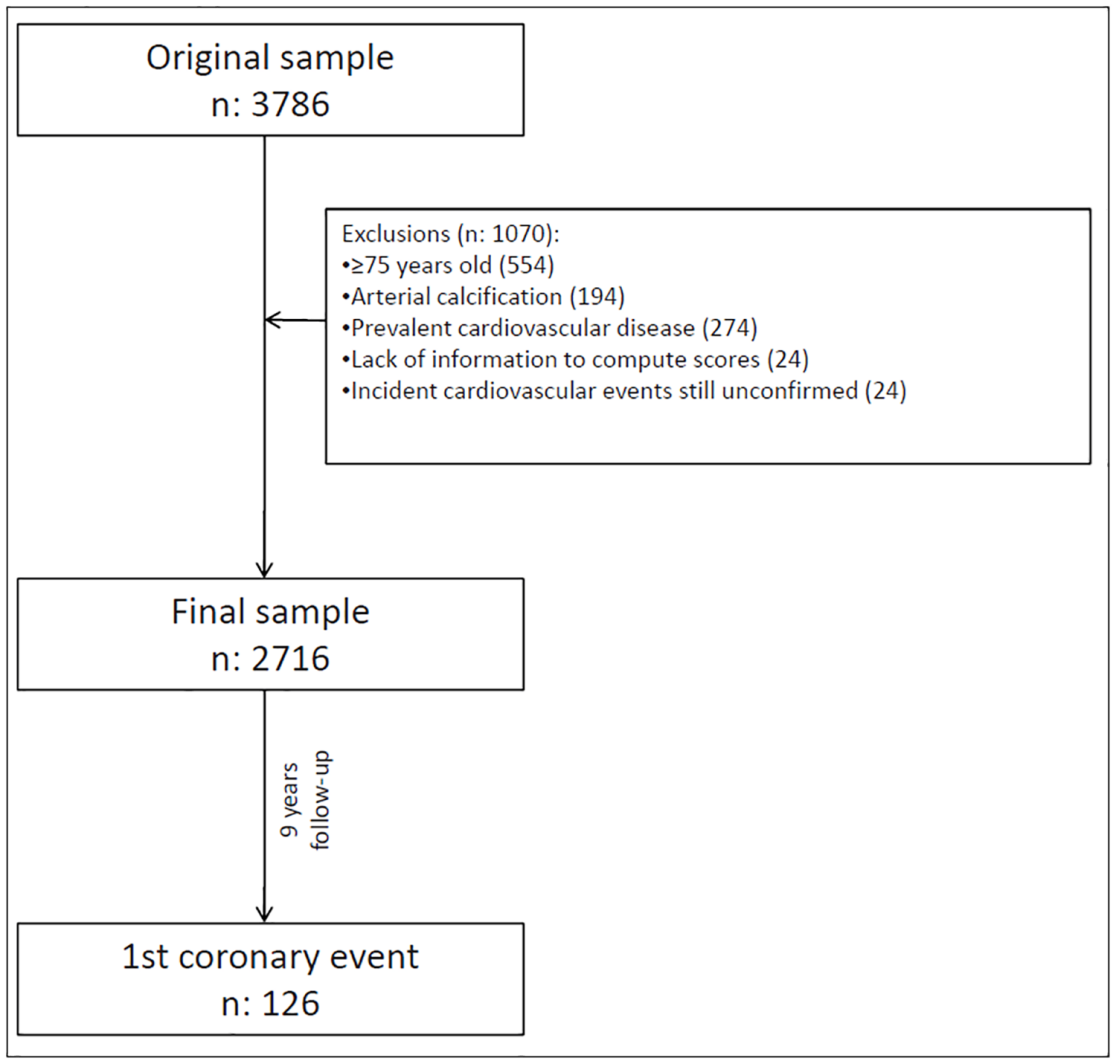
Study flow-chart.

Of the 3,786 participants recruited at the beginning of the study, 2716 were finally analyzed for the evaluation of the reclassification of cardiovascular risk. Fig 1 shows the exclusion criteria.

Table 1 shows the basal characteristics of the study population based on the presence of pathological or normal ABI and the incidence of first cardiovascular events. Of the total cohort, 57% were women with a mean age of 62 years (range 49–74). Pathological ABI was presented by 131 individuals (4.8%). Of the different variables studied these patients were older, 60% were men with a greater proportion of current and former smokers, hypertension, diabetes mellitus and hypercholesterolemia. They also showed a higher cardiovascular risk for the Framingham ≥ 20 and REGICOR ≥ 10 scores. The mean length of follow up was 9 years during which there were 126 cases of first coronary events (5%) and 61 cases of primary cerebral events (2%). The incidence of coronary events in patients with pathological ABI was 4-fold that of subjects with a normal ABI (17.2/1,000 persons-year versus 4.8/1,000 persons-year). The same was observed with the incidence of cardiovascular events (including transient ischaemic attack and stroke), although this was lower in both cases (8.3/1,000 persons-year versus 2.5 /1,000 persons-year).Table 1.

**Table 1:** Sample characteristics (n = 2716).

	Overall		ABI≥0.9 (n = 2585)		ABI<0.9 (n = 131)		p
**Baseline**							
Age	62	7	62	7	65	7	<0.001
49–59 years	1146	42%	1108	43%	38	29%	<0.001
60–69 years	1138	42%	1086	42%	52	40%	
70–74 years	432	16%	391	15%	41	31%	
Women	1557	57%	1504	58%	53	40%	<0.001
Tobacco smoking							<0.001
Never smoker	1536	57%	1492	58%	44	34%	
Former smoker	646	24%	606	23%	40	31%	
Current smoker	534	20%	487	19%	47	36%	
Body mass index							0.017
<25 Kg/m2	486	18%	451	17%	35	27%	
25–30 Kg/m2	1246	46%	1197	46%	49	37%	
≥30 Kg/m2	980	36%	933	36%	47	36%	
Diagnostics (based on medical records)							
Arterial hypertension	1091	40%	1021	39%	70	53%	0.002
Hypercholesterolemia	1229	45%	1155	45%	74	56%	0.008
Diabetes	361	13%	326	13%	35	27%	<0.001
Cardiovascular risk							
Framingham	14	10	14	9	21	13	<0.001
<10%	1078	40%	1049	41%	29	22%	<0.001
10–20%	1025	38%	987	38%	38	29%	
≥20%	613	23%	549	21%	64	49%	
REGICOR	5.8	3.7	5.7	3.6	8.3	5.3	<0.001
<5%	1274	47%	1240	48%	34	26%	<0.001
5–10%	1080	40%	1022	40%	58	44%	
≥10%	362	13%	323	12%	39	30%	
**Follow-up**							
Cardiovascular events incidence							
Follow-up (years)	8.9						
Person-years	24153						
Cardiac events (AMI/angor/revascularization)	126	5%	108	4%	18	14%	<0.001
Cardiac events incidence (x1000py) CI95%	5.3	4.4–6.4	4.8	3.9–5.8	17.2	10.2–27.1	<0.001
Cerebrovascular events (stroke/TIA)	66	2%	57	2%	9	7%	0.001
Cerebrovascular events incidence (x1000py) CI95%	2.8	2.1–3.5	2.5	1.9–3.2	8.3	3.8–15.8	0.001

Result shown as mean and standard deviation or frequency and percentage unless otherwise stated.

Missing values: body mass index (4).

ABI: ankle-brachial index

Table 2 shows the percentage of individuals who were reclassified as having high risk on including the presence of ABI < 0.9 to the levels of low and intermediate risk for each scale, raising the number of subjects at risk by 6% for the Framingham and 8% for REGICOR.

**Table 2:** Cardiovascular risk reclassification adding ABI to the risk tables.

	ABI<0.9		% reclassified as at risk	95%CI	
	No	Yes			
Framingham					
<10%	1049	29	2.7%	1.8%	3.8%
10–20%	987	38	3.7%	2.6%	5.1%
≥20%	549	64	-		
REGICOR					
<5%	1240	34	2.7%	1.9%	3.7%
5–10%	1022	58	5.4%	4.1%	6.9%
≥10%	323	39	-		

ABI: ankle-brachial index

As for the difference of genders 5.3% of men and 6.6% of women are reclassified to high cardiovascular risk when adding ABI to the Framingham scores, and 8.2% and 7.2% are when adding ABI to the REGICOR scores.

**Fig 2:**
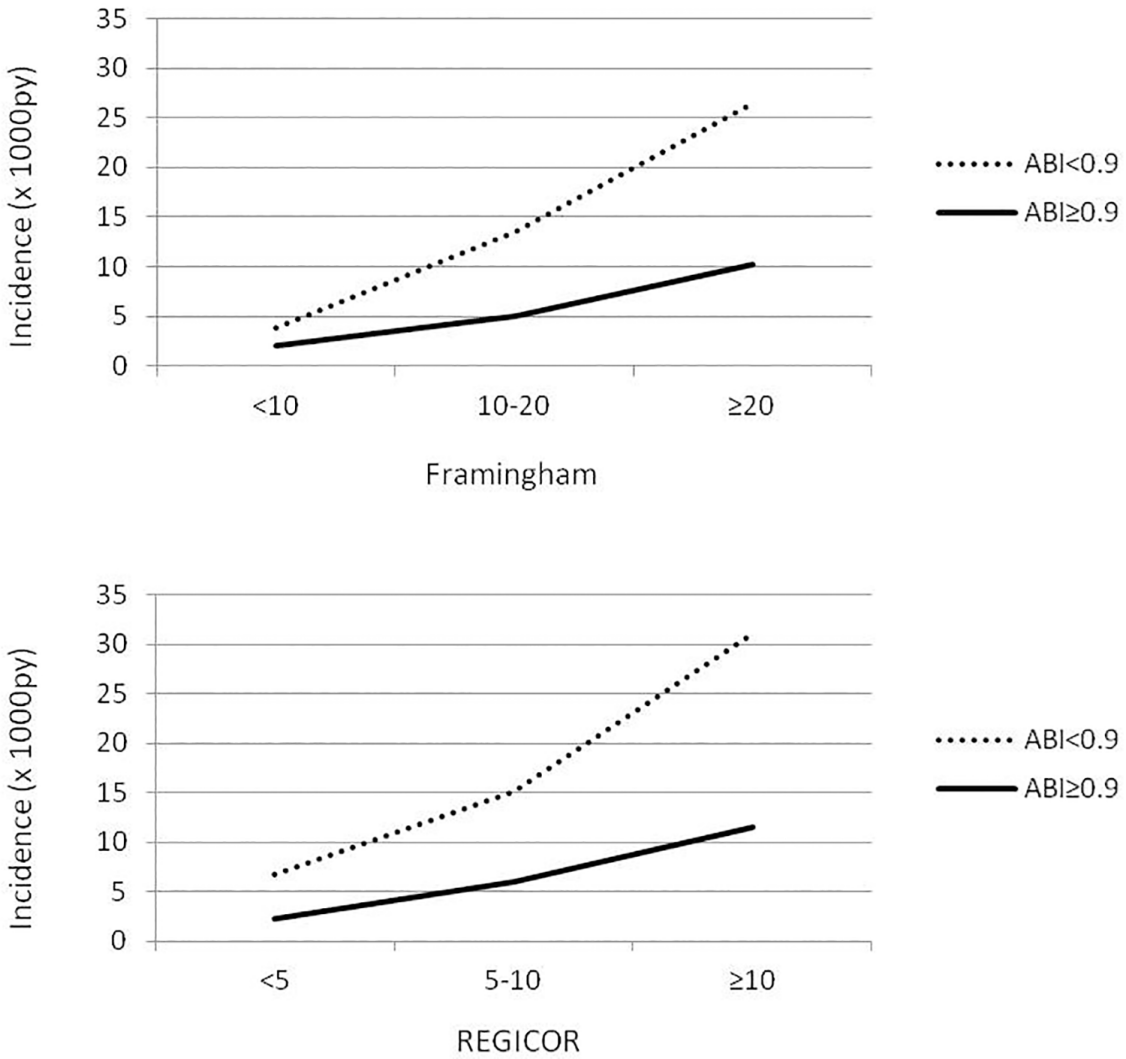
Incidence (x1000py) of coronary events by risk tables and pathological ABI. p-values for PAD-risk table interactions are 0.951 and 0.978 for Framingham and REGICOR respectively.

The incidence of coronary events based on pathological ABI and the risk tables are shown in Fig 2.

According to the two scales, for the same grade of risk the incidence of events was always greater in the patients with pathological ABI. There was no statistically significant interaction between ABI and the risk scales. The adjusted risk of presenting the events of interest and pathological ABI is shown in Table 3.

**Table 3:** Hazard ratio (HR) of having a coronary event using pathological ABI and the risk table category as mutually adjusted explaining variables.

Model	HR	95%CI		p
Framingham+ABI				
ABI<0.9	2.55	1.53	4.24	<0.001
10–20	2.53	1.50	4.27	0.001
≥20	5.19	3.11	8.68	<0.001
REGICOR+ABI				
ABI<0.9	2.65	1.60	4.40	<0.001
5–10	2.62	1.66	4.13	<0.001
≥10	5.09	3.09	8.37	<0.001

ABI: ankle-brachial index

Pathological ABI showed an independent effect with the HR, being greater than that calculated as intermediate risk for the Framingham and REGICOR scales (HR: 2.55 and 2.65 respectively). Table 4 shows the improvement in the predictive capacity in the model described with the inclusion of ABI. The indexes of risk improved in the two scales on including ABI, obtaining NRI values of 4% (95% CI -2%-11% for the Framingham + ABI and 7% for REGICOR + ABI (95% CI 0%-13%). Similar results were found when computing the NRI among only moderate or only low risk individuals.

**Table 4:** Improving of the predictive capacity of the cardiovascular risk tables when including pathological ABI in the models.

	Framingham	Framingham+AP			REGICOR	REGICOR+AP		
Calibration: Akaike Index Criteria	1919	1910			1923	1913		
Discrimination: Harrell’s C	0.68	0.69			0.67	0.68		
Reclassification: NRI			95%CI				95%CI	
Among cases		0.07	0.01	0.14		0.10	0.04	0.17
Among non cases		-0.03	-0.04	-0.02		-0.04	-0.04	-0.03
Overall		0.04	-0.02	0.11		0.07	0.00	0.13
Among cases (moderate risk group)		0.09	0.01	0.16		0.12	0.04	0.20
Among non cases (moderate risk group)		-0.03	-0.05	-0.02		-0.05	-0.06	-0.04
Overall (moderate risk group)		0.05	-0.03	0.13		0.07	-0.02	0.15
Among cases (low risk group)		0.05	-0.05	0.15		0.07	-0.02	0.17
Among non cases (low risk group)		-0.03	-0.04	-0.02		-0.03	-0.03	-0.02
Overall (low risk group)		0.02	-0.07	0.12		0.05	-0.05	0.15

ABI: ankle-brachial index; NRI: net reclassification index. Moderate risk: Framingham between 10–20%, REGICOR between 5–10%.

NRIs for men and women were, respectively, 11% and 0% when adding ABI to Framingham and 10% and 2% when adding ABI to REGICOR, in both cases with overlapping confidence intervals.

## Discussion

Several scales are available to predict cardiovascular events; however, there continue to be difficulties in identifying the individuals most susceptible to presenting these events. In our study it was found that in a low cardiovascular risk setting such as ours an evaluable number of patients may be reclassified as having a category of high risk on adding the pathological ABI to the Framingham and REGICOR scales. Indeed, the ABI is a valid, reliable easy to perform and well accepted test in primary care. The improvement in the reclassification was demonstrated by a global NRI of 4% and 7%, respectively, although the first didn’t reach statistical significance.

The results of this study also confirm the greater incidence of coronary and cardiovascular events in patients with ABI<0.9. There was a notable association between pathological ABI and the incidence of coronary events. HR of having a coronary event using pathological ABI and the risk table category as mutually adjusted was 2.55 in case of Framingham and 2.66 in case of REGICOR. It was similar to the previous results of national [12,18,34] and international [15,19,20,22] studies.

The different guidelines and consensus differ with regard to the recommendations for the use of the ABI in the evaluation of cardiovascular risk. While some recommend the addition of the ABI or consider this index to be a modifier of risk, others are not so favorable or do not make any recommendation in this respect [35–38]. Several studies have also analyzed the reclassification capacity of coronary events with the addition of the ABI to risk scales [7,16–18,20,22,23]. Some of these studies did not observe any improvement in the reclassification of global risk. In the ARIC study [16] the NRI of men and women together was 0.8%, being 0.6% in the Rotterdam study [7] and 1% in the Velescu *et al* study [18] with the use of this statistic. A modest improvement was described with a NRI of 3.3% in the Rodondi *et al* study [22]. Ours results showed a better capacity of reclassification with a global NRI of 7% for the REGICOR+ ABI and 4% for the Framingham + ABI although in this case it was not statistically significant. These results are similar to those of the study by Fowkes *et al* [20] which analyzed 18 cohorts with a total of 44,752 individuals and obtained an NRI of 4.3% in men and 9.6% in women. We have examined the differences between gender in “a priori” risk reclassification and, in our study, they are small. 5.3% of men and 6.6% of women are reclassified to high cardiovascular risk when adding ABI to the Framingham scores, and 8.2% and 7.2% are when adding ABI to the REGICOR scores. Overall figures were 6.4% and 8.0% respectively for Framingham and REGICOR. Some studies have also evaluated the ABI only in patients with intermediate risk and described a better level of reclassification, with the NRI ranging from 5.1 to 23% [7,16,18,20,22]. The NRI results in this group were not statistically significant in our study, but seemed slightly higher than in the low risk patients. Some authors justify the use of the ABI only in the intermediate risk group since a large proportion of the population is within this risk group, and the ABI cannot be determined in all individuals in primary care centers and thus, measurement must be prioritized to a determined group of patients.

There are other scales of risk such as the QRISCK2 [39]; however we were unable to find prospective studies assessing an improvement in reclassification with this tool. This scale includes additional risk factors such as ethnic origin, family history and the level of social exclusion. A recent study [40] undertaken in a population registered in primary care centers in Wales estimated that the use of the ABI does not provide a better reclassification of the calculation of risk with QRISCK2 since this scale itself can predict high cardiovascular risk in most patients with PAD. It is of note that this study was performed in only one centre with a low rate of participation (33%). Moreover, it was a cross- sectional study, and therefore, a larger study with prospective follow up is necessary to obtain more conclusive data.

The prospective 9-year follow up of a population cohort created in primary care is strength of this study. In addition, confirmation of the events was carefully carried out using several methods. The present study provides evidence supporting the use of a model of risk including the ABI in the calculation. The study assessed 2 classical risk scales, the Framingham and REGICOR as they are the most used in our environment.

Possible limitations of the study may be that the conclusions are limited to individuals from 49 to 74 years of age. In our setting, cardiovascular risk is low in patients under this age range, and the prevalence of an ABI < 0.9 is low, and thus, we believe that this did not affect the results obtained. Most validated scales do not include patients over 74 years of age. This is important since the prevalence of pathological ABI as well as the incidence of vascular events markedly increase after this age. Patients with an ABI ≥ 1.4 were excluded because their clinical significance is different compared to patients with a normal ABI.

## Conclusions

The identification of patients at risk of presenting a vascular event should be a priority objective in primary care since it is the level of healthcare with the greatest accessibility to the general population and in which primary prevention strategies can be most adequately implemented. Despite the discrepancies regarding the inclusion of the ABI to scales for predicting cardiovascular risk and even though other parameters provide greater evidence in this regard [4,7,8], an easy to manage tool which can be applied in primary care is needed to improve the sensitivity of the risk scales used. The results of the present study support the addition of the ABI as a tool to help reclassify coronary risk and to confirm the greater incidence of coronary events in patients with pathological ABI in whom early diagnosis is essential.

## Supporting information

S1 File Additional information from the study database. (XLSX)
