# Correction: Contribution of the ankle-brachial index to improve the prediction of coronary risk: The ARTPER cohort

**DOI:** 10.1371/journal.pone.0197139

**Published:** 2018-05-07

**Authors:** Rosa Forés, Maria Teresa Alzamora, Guillem Pera, José Miguel Baena-Díez, Xavier Mundet-Tuduri, Pere Torán

There is an error in affiliation 3 for authors Rosa Forés and Xavier Mundet-Tuduri. Affiliation 3 should be: Departament de Medicina. Universitat Autònoma de Barcelona, Bellaterra (Cerdanyola del Vallès), Spain.
